# Management of Orthodontic Therapy-Associated Gingival Overgrowth for Esthetic Consideration in Anterior Maxillary Region: A Case Report

**DOI:** 10.7759/cureus.63709

**Published:** 2024-07-02

**Authors:** Shivani S Thakare, Unnati Shirbhate, Pavan Bajaj, Anand Wankhede

**Affiliations:** 1 Department of Dentistry, Sharad Pawar Dental College & Hospital, Datta Meghe Institute of Higher Education & Research, Wardha, IND; 2 Department of Periodontics, Sharad Pawar Dental College & Hospital, Datta Meghe Institute of Higher Education & Research, Wardha, IND

**Keywords:** periodontal plastic surgery, ortho-perio, orthodontic induced gingival enlargement, gingival enlargement, external bevel gingivectomy

## Abstract

Gingival inflammation and fibrous type of overgrowth, or a combination of both can lead to gingival enlargement (GE), and this negatively affects mainly masticatory function and esthetics, and sometimes causes psychological issues in patients. A typical characteristic of gingival diseases is gingival overgrowth, which can be brought on by fibrous overgrowth, gingival inflammation, or a combination of the two. It is a complex ailment arising from interactions between the environment and the host or different stimuli. Patients frequently have misaligned teeth, which encourages the buildup of bacterial plaque and unintentionally fuels gingival inflammation. Fixed orthodontic equipment can rectify this misalignment but they may also promote plaque buildup and the ensuing development of GE, gingival invaginations, and generalized hyperplastic gingivitis. The attachment of application and the rise in the amount of discernible supra- and subgingival plaque cause changes in microbial growth. Moreover, the force used in the treatment tends to activate the gingival soft tissue response. Clinical consequences such as persistent infection, inflammatory hyperplasia, gingival recession, attachment loss, or gingival overgrowth may arise after the device is placed. 'Plaque-induced' and 'non-plaque-induced' gingival disorders, such as gingival overgrowth, can be distinguished; however, a more precise fundamental etiology is frequently discernible. Several hereditary, systemic, or infectious diseases do not depend on plaque induction. Accompanying plaque accumulation in certain circumstances may make the clinical appearance worse. The case described here is of a 21-year-old female patient presenting with anterior maxillary GE associated with lateral incisors with orthodontic therapy. Surgical therapy was carried out to provide an excellent esthetic outcome for the patient.

## Introduction

Nowadays, gingival overgrowth (GO), in which the size of the gingiva increases, is a common feature of gingival disease. The accepted current terms for this condition are GO and gingival enlargement (GE). Depending on the extent, the overgrowth impairs the stomatognathic apparatus in various ways, including functional abnormalities (such as speech impairment), chewing difficulties, and esthetic issues. It can also lead to severe psychological problems. Even with all the research that has been done, there are still many unknowns, particularly concerning the etiopathogenic issues [1]. A typical characteristic of gingival diseases is GO, which can be brought on by fibrous overgrowth, gingival inflammation, or a combination of the two. It is a complex ailment arising from interactions between the environment and the host or stimuli [2]. 'Plaque-induced' and 'non-plaque-induced' gingival disorders, such as GO, can be distinguished; however, a more precise fundamental etiology is frequently discernible. Several hereditary, systemic, or infectious diseases do not depend on plaque induction. Accompanying plaque accumulation in certain circumstances may worsen the clinical appearance [3]. Generally, gingival tissues are injured and repaired by a recurrent cycle of inflammatory cell recruitment, chemotactic factor generation, tissue resorption, replacement, and remodeling. The turnover of collagen in periodontal tissues is remarkably high [4]. Patients frequently have misaligned teeth, which encourages the buildup of bacterial plaque and unintentionally fuels gingival inflammation. Fixed orthodontic equipment can rectify this misalignment but they may also promote plaque buildup and the ensuing development of GE, gingival invaginations, and generalized hyperplastic gingivitis [5]. Histological features in these types of patients show the thickened gingival epithelium and connective tissue layer, and more fibrous with dense organization of fiber bundles. This demonstrates that GO brought on by orthodontic therapy has a strong epithelial proliferation response backed by more collagen fiber bundles, comparable to other overgrowth kinds like drug-induced GE [6]. Although the precise process underlying this increase is unknown, it is not always linked to a rise in the quantity or size of fibroblasts. Possible mechanisms including excessive proliferation of epithelial cells and the formation of GO may be related to increased expression of type I collagen mRNA and regulation of the development of keratinocyte growth factor receptors [7]. Placing an orthodontic device in a patient's mouth modifies the quality of the local bacterial biofilm, and it is frequently linked to changes in the patient's oral hygiene and periodontal health behaviors. The literature on the impact of orthodontic treatment has described the rise in supra- and subgingival plaque quantity and the alterations in the microbiological environment related to device installation [8].

The process known as a gingivectomy removes the enlarged gingival tissue's pocket wall. The primary goals of a gingivectomy are to restore normal physiologic gingival contour, provide an environment that promotes healing, and increase accessibility and visibility for comprehensive debridement of plaque and calculus [9]. Gingivectomy with beveled incisions can be performed to remove soft tissues from the facial surface without affecting the papillary tissue, provided that there are sufficient connected gingiva, more than 3 mm of gingival tissues (from bone to gingival crest), and acceptable osseous levels. However, a straightforward gingivectomy that exposes the entire anatomic crown will not be recommended if osseous levels are close to the cementoenamel junction since this could compromise the supracrestal tissue attachment [10].

## Case presentation

**Figure 1:**
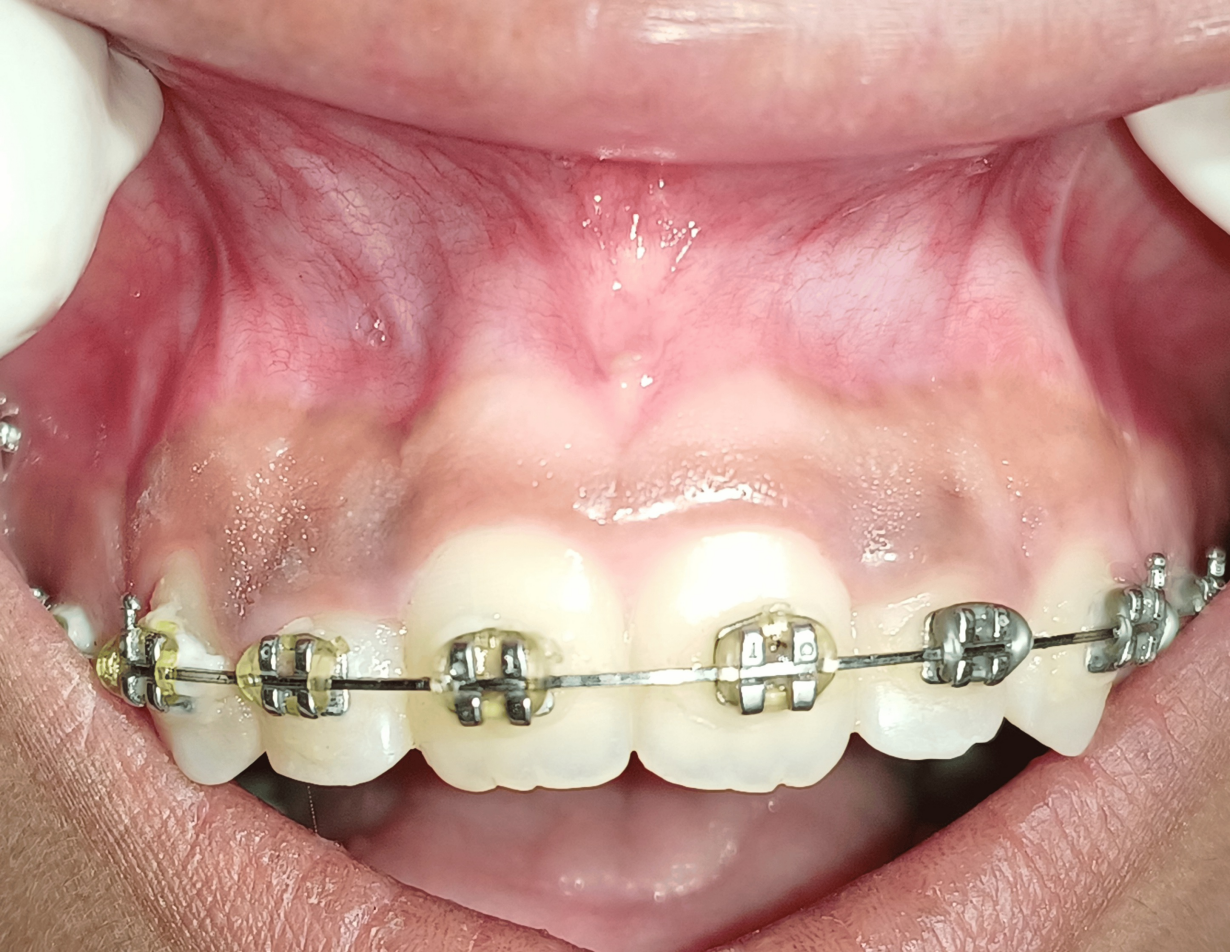
Preoperative view of the patient showing gingival overgrowth associated with lateral incisors

A 21-year-old female patient was referred to the Department of Periodontics from the Orthodontic Department with a chief complaint of GO associated with both right and left maxillary lateral incisors as seen in Figure 1. The patient was advised for scaling and polishing as initial therapy. After a week, the clinical assessment was done, and the overgrowth hadn’t subsided with lateral incisors; therefore, the patient was advised for a gingivectomy procedure with right and left maxillary lateral incisors. Initially, the blood investigations were carried out, which were normal within ranges. The patient's written informed consent was obtained. 

**Figure 2:**
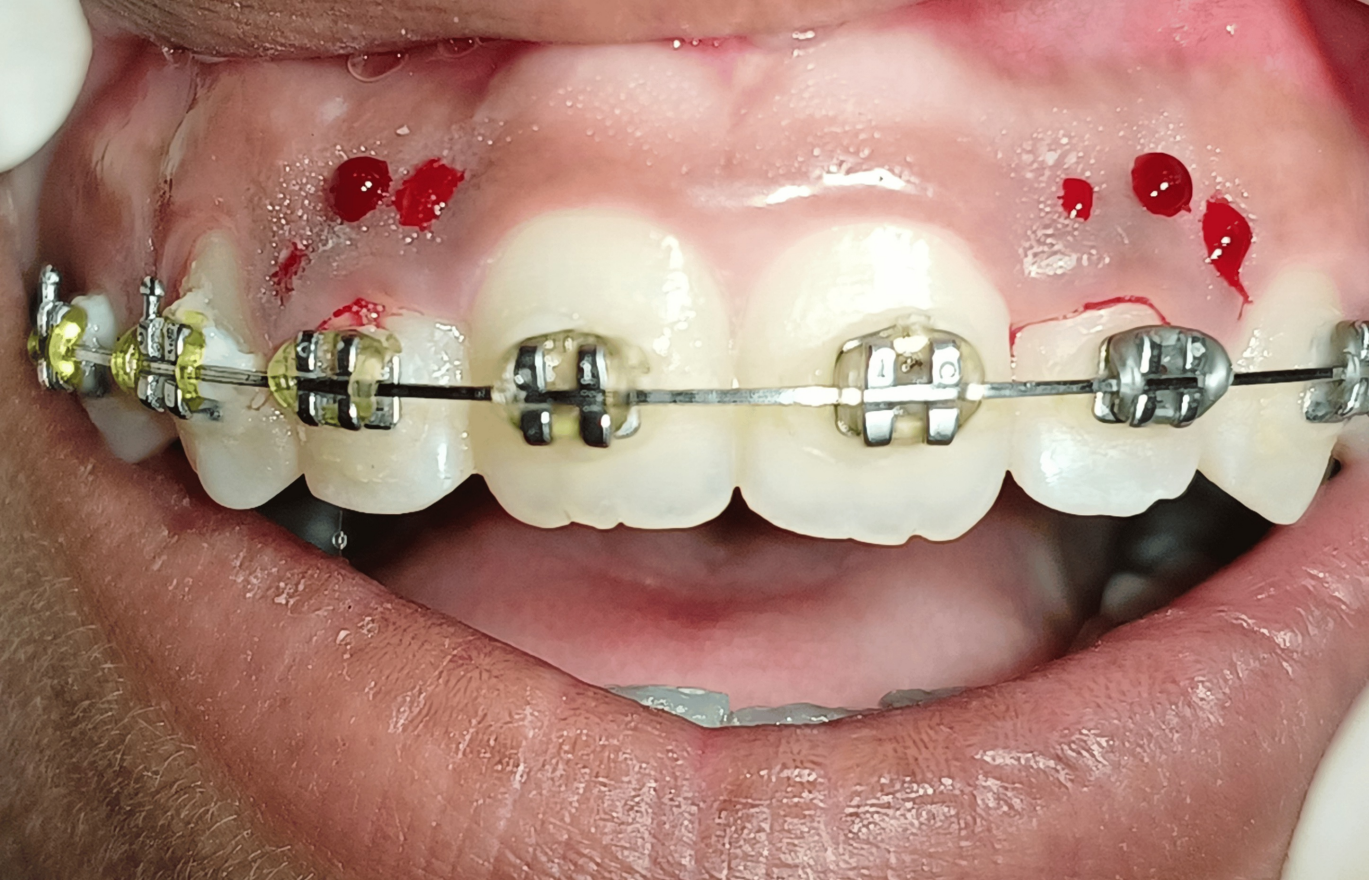
Bleeding points were marked with both lateral incisors

The bleeding points were marked with right and left maxillary lateral incisors with the help of a pocket marker at the deepest position, measuring probing pocket depth as seen in Figure 2. 

**Figure 3:**
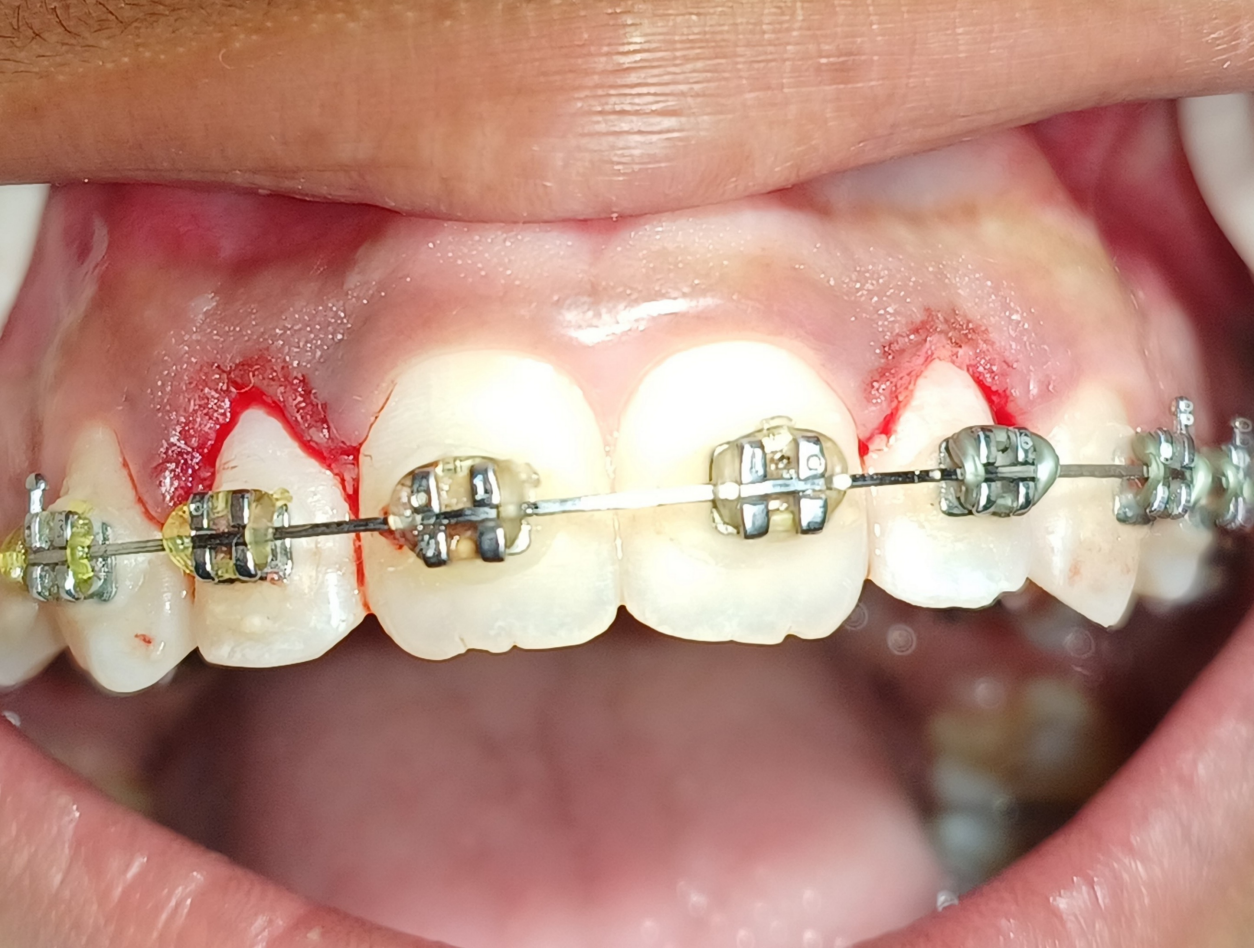
External bevel gingivectomy performed with lateral incisors with the conventional technique

The conventional gingivectomy surgical procedure was carried out using a scalpel by giving an external bevel incision by placing the small curved scalpel blade (number 15) for a precise incision at 45 degrees to the respective tooth, as shown in Figure 3. After completion of the procedure, the hemostasis was achieved. 

**Figure 4:**
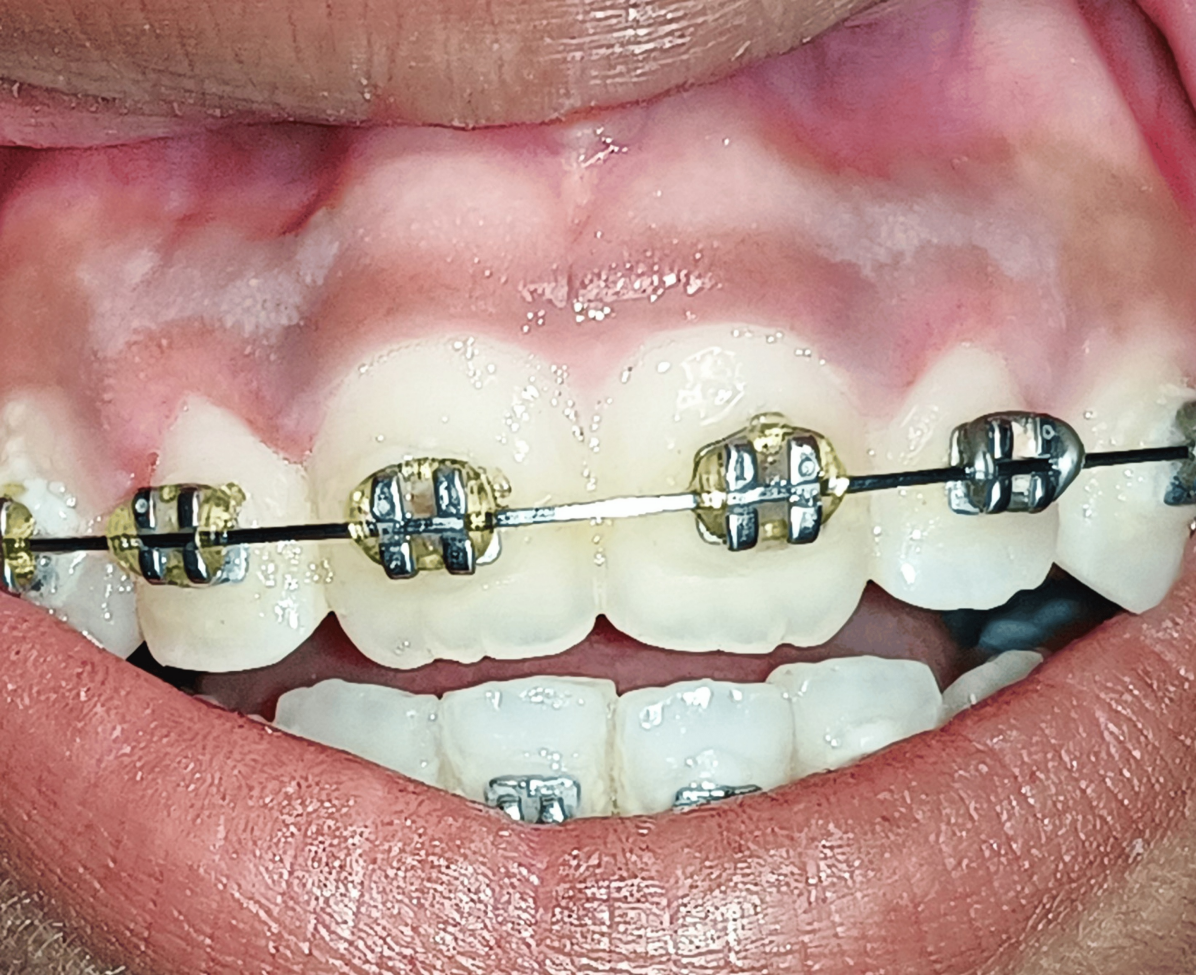
Postoperative view of the patient after one month showing complete satisfactory healing

The patient was reviewed after seven days and one month for further periodontal evaluation which demonstrated satisfactory healing, and no signs of infection or swelling. After one month of follow-up examination, the surgical site demonstrated complete satisfactory healing, no signs of recurrence, and an esthetically admired smile appreciated in Figure 4. 

## Discussion

Since most permanent teeth have erupted by the time an adolescent reaches adulthood and can still be used to drive craniofacial growth, orthodontists typically see this stage of life as supportive of orthodontic therapy. This permits the correction of malocclusions and the displacement of teeth while preserving average facial growth [9,11]. Therefore, this is the time when most orthodontic treatments are started. Even with the systematic instructions on oral hygiene provided at the start of treatment, young patients frequently struggle to maintain good oral hygiene, particularly during adolescence when it can be challenging to get compliance and hormonal fluctuations can exacerbate gingival inflammation [11]. The attachment of application and the rise in the amount of discernible supra- and subgingival plaque cause changes in microbial growth. Moreover, the force used in the treatment tends to activate the gingival soft tissue response. Clinical consequences such as persistent infection, inflammatory hyperplasia, gingival recession, attachment loss, or GO may arise after the device is placed [12]. To prevent GE, educational initiatives should be undertaken to achieve orthodontic therapy with gingival health. Surgical methods are required to promptly treat gingival tissue inflammations that jeopardize the effectiveness of orthodontic treatment when oral hygiene care is still inadequate. However, as surgical gingivectomy was ineffective in the long-term monitoring if self-care was reduced, clinical significance should be interpreted cautiously [13]. In addition to having a negative impact on periodontal health and esthetics, excessive gingival tissue can also impair masticatory function by reducing the protective barrier against trauma. The majority of patients receiving orthodontic treatment are younger people who are more self-conscious about their appearance, and gingival hyperplasia is an unsightly condition [12,13].

A gingivectomy is required when the gingiva has torn away from the teeth, leaving deep pockets. It is challenging to remove calculus and plaque from the pockets. Usually, a gingivectomy is performed before gingival disease, causing damage to the tooth-supporting bone. The loose, unhealthy gingival tissue is removed and reshaped during the operation to eliminate spaces between the teeth and the gingiva. Gingivectomy creates visibility and accessibility for removing calculus and smoothing the tooth roots by eliminating the pocket walls. Doing so creates an environment conducive to gingival healing and gingival contour restoration [14].

## Conclusions

Gingivectomy is currently a widespread cosmetic surgery despite its original development as a treatment for periodontal disease. It is applied to eliminate excessive gingival tissue and enhance the esthetics of the gingiva. This case report demonstrates that the conventional gingivectomy aspect in orthodontic patients fulfills the esthetic as well as the functional demands of the patient. The orthodontic and periodontic relationship fixes the treatment outcomes and the esthetic demands associated with orthodontic therapy in GE.
